# SIENA: Semi-automatic semantic enhancement of datasets using concept recognition

**DOI:** 10.1186/s13326-021-00239-z

**Published:** 2021-03-24

**Authors:** Andreea Grigoriu, Amrapali Zaveri, Gerhard Weiss, Michel Dumontier

**Affiliations:** 1 Institute of Data Science, Maastricht University, Universiteitsingel 60, Maastricht, 6229 ER Netherlands; 2 Department of Data Science and Knowledge Engineering, Maastricht University, Paul-Henri Spaaklaan 1, Maastricht, 6229 EN Netherlands

**Keywords:** Ontology, Semantic enhancement, Gene, Deep learning, Machine learning

## Abstract

**Background:**

The amount of available data, which can facilitate answering scientific research questions, is growing. However, the different formats of published data are expanding as well, creating a serious challenge when multiple datasets need to be integrated for answering a question.

**Results:**

This paper presents a semi-automated framework that provides semantic enhancement of biomedical data, specifically gene datasets. The framework involved a concept recognition task using machine learning, in combination with the BioPortal annotator. Compared to using methods which require only the BioPortal annotator for semantic enhancement, the proposed framework achieves the highest results.

**Conclusions:**

Using concept recognition combined with machine learning techniques and annotation with a biomedical ontology, the proposed framework can provide datasets to reach their full potential of providing meaningful information, which can answer scientific research questions.

## Background

The amount of data becoming available is rapidly increasing. Various research fields can benefit from the growing volume of information, including the biomedical domain. Unfortunately, answering a research question using the already available data usually requires information which can be found in more than one dataset. Moreover, the information needed is not only spread across sources, but also is stored in different formats such as comma-separated values (CSV), extensible markup language (XML) etc. Therefore data processing is usually needed to solve the provided task. However, data processing has been identified by 80% of data scientists as the most time consuming part of a project and at the same time, the least enjoyable one [1].

In response to this demand, many tools involving various types of data integration and conversion are being developed. Data2Services [2] is such a tool that provides an automatic conversion of various datatypes to the Resource Description Framework (RDF)^1^ format, which can help with data integration. The RDF format provides a structured, standardized and machine readable data representation.

However a structured format does not necessarily provide meaning to the data. For data to be meaningful and understandable, additional information, such as knowing what the columns of the dataset represent (their types) and how they are related (interoperability), is required. To semantically enhance the data, one could annotate the data with existing concepts, in the form of public ontologies.

**Fig. 1:**
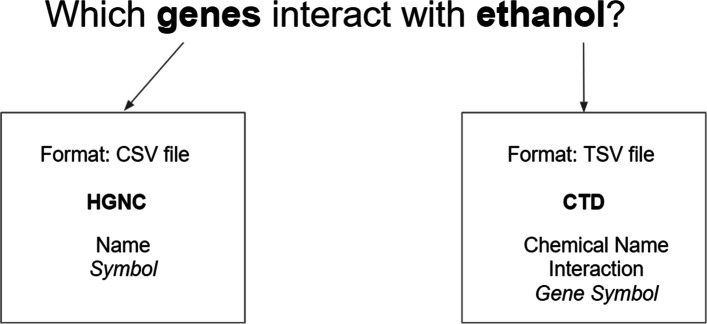
Use case. Figure is showing a research question use case, where the answer is found in two different datasets

As a use case, consider the following query that a biomedical researcher is interested in: *Which genes interact with ethanol?*, in order to know how ethanol, that could be used as a component of a drug, reacts with human genes. The answer to this question already requires using two separate datasets, namely Hugo Gene Nomenclature^2^, for gene information, and Comparative Toxicogenomics Database^3^, for information about ethanol. These datasets are available in two different formats CSV, and tab-separated values (TSV), respectively. The two sources share common data attributes, such as gene symbol, and common data values such as the indexed genes. However the gene symbol attribute is represented using two different labels : “Symbol”, “Gene Symbol”. This is represented in Fig. 1.

Without data integration, this would be solved through manual analyses of the data and extraction of the correct answer, which can be a time consuming process. Combining the two datasets can provide the answer. Data2Services can make both datasets publicly available in a common format. However, the tool provides generic transformation of the data. Therefore, a manual investigation is still needed to determine that the two columns containing symbols represent the same attribute, (see Fig. 1), therefore having the same meaning. This can be solved through semantic enhancement. If data would also be semantically annotated, the two columns should be sharing the same concept.

Therefore, this project is addressing the following research question: *Can we (semi-)automate the transformation of biomedical datasets into a semantically meaningful representation?*, specifically addressing if we can automatically assign the concept for a column label in a tabular data file. In this project, we only focus on gene datasets.

This project has the following contributions:

- methodology of using a public biomedical ontology repository to identify relevant gene concepts
- developing two separate methods for gene concept recognition through machine learning classification
- implementation of a framework performing semi-automatic semantic enhancement using the explored methods
- report of quality assessment of the resulting data

There are different tools that can provide RDF conversion from multiple data types [3–7]. However, they require considerable amount of human input. Data2Services [2] can automatically convert different data formats (e.g. CSV, XML) to RDF. However, it provides a generic outcome missing out on semantic types for entities and their relations.

Ontology mapping tools help users map ontology terms to their data. However, in most tools, the user needs to provide the ontology that will be used for the mapping [8–10] or chose from the recommended options [11].

In [9], the task of concept recognition in biomedical data is defined as mapping a piece of text to a previously selected terminology (or in some cases an ontology). Two concept recognition tools are compared in [9], using different dictionaries and data as input. The data mostly contains free text. The results show that the performance varies with different data as input and dictionaries. Therefore, good performance of those concept recognition tools is linked to the prior selected dictionary and dataset. Other approaches combine machine learning techniques such as classifiers into the mapping process [12]. However, using pre-selected dictionaries and free text input data restrain the data and concepts that can be explored. In order to preserve the semantic characteristics of words (linguistic meaning), low-dimensional vectors such as word embeddings can be used as word representations, which have proven to be effective in various tasks [13, 14].

This paper introduces a concept recognition task using machine learning, specifically binary classification, used for semi-automated semantic enhancement of data. In our experiments, we have focused on gene datasets, so the gene concept. However, our method does not depend on pre selected data and/or preselected dictionaries as explored in previous papers. In addition, our approach uses word embeddings on a dataset with heterogeneous values, therefore, the input data is also no longer limited to free text.

## Methodology

**Fig. 2:**
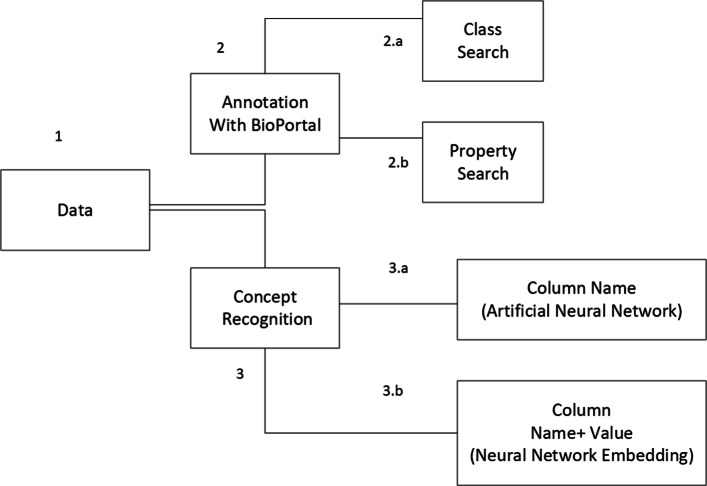
Methodology diagram. The data is used for search in BioPortal in order to provide data annotation, but also pre-processed to be used as input for the concept recognition task

We investigated two approaches: (i) annotation with BioPortal and (ii) concept recognition. We developed a framework combining both to tackle the problem of semi-automatic semantic enhancement. Figure 2 presents an overview of the applied methods.

The project focuses on providing semi-automated semantic enhancement to three datasets (Hugo Gene Nomenclature, Comparative Toxicogenomics Database and Pharmacogenomics Knowledgebase) which are automatically converted to RDF using the Data2Services tool.

The first method, described in “Annotation with BioPortal” section aims to solve the task of semantic enhancement by using a biomedical ontology repository. The repository can provide both types (classes) and attributes (properties) for the searched term, through separate search options. However, the results might differ for each type of search. For example, the term “Chemical Id” has no matches in a class search, in contrast to 30 matches in a property search. Two separate experiments are conducted in order to establish whether the method should be used to provide properties or classes for the task.

The second method, described in “Concept recognition model” section focuses on automatically recognizing the presence of a class in a dataset. We define “Concept recognition” section as a task where we determine if the gene concept is present in a dataset using binary classification. We developed two separate approaches. The first approach uses the combination of column names (titles) presented in a dataset and the corresponding values (data) in the columns as input for the concept recognition task. In the second approach, only the column names (titles) are used as input for the same task.

The following sections describe the method components in detail. “Datasets” section describes the data used, “Annotation with BioPortal” section focuses on the use of a biomedical ontology repository and “Concept recognition model” section defines the developed concept recognition method.

### Datasets

In order to determine the performance of the chosen methods on a smaller scale first, a small corpus sample was chosen. We chose three datasets: (i) Hugo Gene Nomenclature (HGNC), (ii) Comparative Toxicogenomics Database (CTD) and (iii) Pharmacogenomics Knowledgebase (PGKB).

- HGNC^4^ is a publicly available database which contains all the curated HGNC approved nomenclature, gene groups and associated resources. This project uses the complete HGNC dataset file.
- CTD^5^ is a publicly available database which contains manually curated information about chemical–gene/protein interactions, chemical–disease and gene–disease relationships. The subset containing chemical–gene/protein interactions was chosen for this project.
- PGKB^6^ is a pharmacogenomics knowledge dataset that incorporates various curated clinical information such as dosing guidelines and drug labels, potentially clinically actionable gene-drug associations and genotype-phenotype relationships. We used the subset containing gene information used by PGKB.

Further details about the data are presented in Table 1.

**Table 1:** Detailed information about the datasets used in the methodology

Data	About	Subset	Size	Column Number	Column Name
HGNC	Standardized nomenclature to human genes	Subset of complete HGNC	382 KB	49	symbol, locus type, ena
CTD	Manually curated information	Chemical–gene interactions set	326 MB	11	Chemical ID, Gene Forms,
					PubMed IDs
PGKB	Information about how human genetic	Summary of the gene information	13.6 MB	17	Ensemble Id, Chromosome,
	variation affects response to medications				Cross-references

These particular datasets were chosen due to their focus on the gene terminology. The data describes different aspects related to the gene concept such as names, symbols and different identifiers provided by official organizations such as Hugo Gene Nomenclature, National Center for Biotechnology Information. Apart from these commonly found elements, each dataset contains specific elements of topics such as chemical identifiers and interactions (chemical-gene interaction).

### Annotation with BioPortal

BioPortal^7^ is a repository of biomedical ontologies. Together with the meta-data, the contained classes and properties are publicly available for each ontology [15]. There are 729 ontologies relevant for the biomedical domain. Ontologies can be browsed via an interface or accessed via an API^8^ to query using a variety of parameters (e.g providing a restricted ontology list, retrieving exact matches of the searched term). Therefore, we used the BioPortal annotator for our project, illustrated in part 2 of Fig. 2. We used the column names (titles) as search terms in BioPortal. Considering the available search options in BioPortal, we conducted two separate search types: (i) class search and (ii) property search. The experiments were executed on a laptop^9^ which imposed a restriction in doing them at a big scale (BioPortal has over 9 million classes).

#### Class search

Since we performed the experiments at a small scale, we restricted the class search by using the parameter longest matches (LM). This parameter returns matches only if the full searched term is found in the matched class in BioPortal.

#### Property search

The unrestricted property search generated an infeasible amount of matches to be analyzed. For each dataset, the column name with the highest amount of matches from the property search can be observed in Table 2. In total, there were 25 columns which had over 1000 matches.

**Table 2:** Examples of high numbers of property search matches per dataset

Dataset	Columns	Matches
CTD	Gene Id	1681
PGKB	Has Variant Annotation	15923
HGNC	date name changed	2075

Therefore, to restrict the number of results, we chose to provide a list of relevant ontologies for the search. To choose the most relevant ontologies, we selected the ones that were most popular (had the highest amount of matches) in the initial search. We computed a frequency distribution of the number of matches, by counting the number of matches per ontology, for each dataset. We built the distribution based on the initial matches that resulted from the unrestricted property search. We observed that there are fewer ontologies with a high number of matches, with most having around three matches. The list of ontologies is filtered such that only the top most popular remain by choosing a threshold represented by the mode of the distribution, which is different for each dataset. (e.g. at least eight matches per ontology in the case of the CTD dataset).

A threshold value of 8 was chosen for CTD, 30 for HGNC and 12 for PGKB. This analysis was needed due to high number of ontology matches:475 unique ontologies for CTD, 561 unique ontologies for HGNC and 593 unique ontologies for PGKB. Therefore, we created a representative top of ontologies using the threshold restriction.

After evaluating the number of matches that each of these ontologies have, a shorter list of ontologies was chosen for the search restriction, according to the highest number of matches present in the graph. A list of three ontologies was chosen for the HGNC and PGKB dataset. CTD had a special situation where only 2 ontologies were distinctive in their number of matches, the rest having the same number. Two ontologies, Neuroscience Information Framework Standard Ontology (NIFSTD) and Orthology Ontology (ORTH), are shared by two datasets, PGKB and CTD.

A property search was conducted using BioPortal, using as a restriction a list of ontologies to be used in the search, which were discovered in the process described above. The resulting matches were manually analyzed in order to establish if they are relevant (have the same semantic meaning as the column label). The matches were classified into three categories to show how semantically relevant they are to the searched term:

- “Full Match” consists of terms that have an exact name match with the column name, or terms which have a description that is appropriate for the column. (e.g. “Horde_ID” for the column ‘horde_id’ of the HGNC dataset)
- “Semi-Match” contains properties that have common terms in the property’s name and meaning with the column name, although it does not define the exact same relation.(e.g. “id” for the column “horde_id” of the HGNC dataset; the “id” match can be used to represent an id relation, but it does not point to a specific id type like the column name “horde_id”)
- “No Match” represents properties that are completely unrelated with the column name. (e.g.“GDB_ID _mapped_data_” for the column “horde_id” of the HGNC dataset)

### Concept recognition model

As we focused on gene datasets, we aimed at recognizing the gene concept in a dataset. We chose a binary classification approach. We chose two separate approaches for the binary classification. The first approach presented, “Column name approach” section, aims to recognize a concept only by the column names(titles) used by a dataset. In contrast, in the second approach, “Column name and value approach” section, we use both the column name and the values found in respective the column to recognize the concept.

#### Column name approach

As the first approach focuses on using only column names (titles), the amount of names offered by the data were insufficient for a successful machine learning approach. We extracted a total of 93 names from the three datasets. Therefore, to perform binary classification and to provide the machine learning algorithm sufficient examples to learn from, we needed to expand the total number of column names. Two separate processes of expanding the list of names were applied: (i) heuristic expansion and (ii) Recurrent Neural Network (RNN) [16] generation expansion. This decision was taken in order to be able to preserve the consistency between both approaches by giving them the same starting point, the same three datasets.

The heuristic expansion was developed in five steps:

- generate all the existing titles in lowercase characters(e.g.:“HGNC ID”)
- generate all the existing titles in uppercase characters(e.g.:“hgnc id”)
- generating title by replacing the white space with underscores(e.g.:“hgnc_id”)
- generating titles by replacing the underscores with white space(e.g.:“hgnc id”)
- splitting all strings separated with underscore or white space and their random re-concatenation (e.g.: column titles “gene family” and “prev symbols” are split into a list [“gene”, “family”,“prev”, “symbol”] and by random re-concatenation we can get the string “symbolgene”)

In total, 1074 column names were generated.

The second process continues the expansion of the obtained list of names using RNN. Since we had a list of words, character level RNN was applied, its type being the most appropriate in this case. Manual class labelling was needed to prepare the data for a binary classification task. The examples that resembled the original names of the column were labelled as positive. The strings that were incoherent, just formed by a random order of characters were labelled as negative examples. A total of 2135 names were obtained after applying the RNN generation. We converted the names into vectors, in which each character of a name was represented by its ASCII code. All vectors have the same size, the size of the vector being determined by the longest string in the list of names. The ASCII code was used to convert each character into a numerical value that was inserted into the vector. In the case of the string being smaller than the vector size, the vector is filled with 0 until it reaches the required size. The resulting matrix was split into a training and test set. The training set matrix was used as training input for an Artificial Neural Network (ANN).

#### Column name and value approach

In the second approach we use both the column names, which were used in the previous approach, and the values (content) of the columns as data. This approach begins by applying a data preprocessing method on the data. In order to be processed by any machine learning technique, the data needs to be transformed into a numerical format. Apart from the difficulty created by the existing diversity in the column name types (e.g. full words, acronyms, short versions of words etc.), the content of the columns (data values) generate obstacles as well. Data values can range from words, numerical values to a mix in between (symbols). The following method was inspired by an existent approach^10^.

A sample of 17 columns (presented in Table 3) out of 93 in total were chosen from all three datasets. Several columns are common across the datasets, therefore they were chosen as part of this sample as positive examples. These columns are considered distinctive for the gene concept (e.g. hgnc id, gene symbol). The columns which were not common throughout the datasets were considered negative examples (location sortable, date). Pairs of the form “column name, data” (e.g [symbol,A1BG]) were constructed with all the chosen columns. For each column name, the number of pairs was given by the size of that particular column. Each column name was assigned a numerical value from 0 to 16.

**Table 3:** Data used for neural network embedding

Column	Dataset	File	Class Label
hgnc id	HGNC	hgnc.tsv	1
HGNC id	pgkb	genes.tsv	1
Name	HGNC	hgnc.tsv	1
name	PGKB	genes.tsv	1
symbol	HGNC	hgnc.tsv	1
Symbol	PGKB	genes.tsv	1
Gene Symbol	CTD	CTD_chem_ gene_ixns.csv	1
location	HGNC	hgnc.tsv	0
location sortable	HGNC	hgnc.tsv	0
date approved reserved	HGNC	hgnc.tsv	0
date modified	HGNC	hgnc.tsv	0
Chromosome	PGKB	genes.tsv	0
Chromosomal Start - GRCh37.p13	PGKB	genes.tsv	0
Chromosomal Stop - GRCh37.p13	PGKB	genes.tsv	0
Chromosomal Start - GRCh38.p7	PGKB	genes.tsv	0
Chromosomal Stop - GRCh38.p7	PGKB	genes.tsv	0
PharmGKB Accession Id	PGKB	genes.tsv	0

Two different methods were used to index the data values. The values that belong in the same category (e.g. gene symbols) are indexed within the same range (e.g from 0 to 200). However, the values that belong into a different category (e.g. gene names) start the indexing at 0. The second method, using character-level RNN, continues increasing the indexation number when handling different categories.

The machine learning model chosen to perform the classification is a Neural Network with embeddings (NNE). The embeddings are represented by the weights of the network which are adjusted during training. Two parallel embedding layers map the column and the value to vectorial representations. Table 4 contains the details of the NNE.

**Table 4:** Structure of the neural network with embeddings

Layer	Output Shape
Columns (Input Layer)	(None,1)
Data (Input Layer)	(None,1)
Column Embedding	(None, 1, 50)
Data Embedding	(None, 1, 50)
Dot product	(None, 1, 1)
Reshape	(None, 1)
Dense	(None, 1)

At first, the NNE was trained with all the data available (145050 pairs) in order to test the quality of the embeddings, through a similarity measure between columns. Table 5 shows the most similar columns that were recommended for the column “hgnc_id” using the first method. Even though the similar recommended columns might seem correct, there were inaccuracies identified in the semantics such as column“symbol” (e.g.“AABT”), representing the approved gene symbol by HGNC, treated as being identical to “hgnc_id”(e.g“12”),representing the unique id created by HGNC to a particular symbol. Table 6 shows the same example, using the second method, where the two most similar recommended columns are hgnc id’s (column “HGNC Id” is considered almost identical with a 99% similarity). Therefore, the second method is more semantically accurate since it can identify columns that represent the same attribute (hgnc id), and can differentiate from other attributes (such as symbol which only has a 67% similarity).

**Table 5:** Method 1 Similarity results: Top most similar columns with “hgnc_id”

Columns	Similarity
hgnc_id	1.0
symbol	1.0
Name	0.99
name	0.99
HGNC Id	0.98
Symbol	0.97
Gene Symbol	0.94
Chromosomal Start p.13	0.58
Chromosomal Stop p.13	0.57

**Table 6:** Method 2 Similarity with continuous indexation: : Top most similar columns with “hgnc_id”

Columns	Similarity
hgnc_id	1.0
HGNC Id	0.99
Symbol	0.67
Gene Symbol	0.67
Symbol	0.66
Chromosomal Start p.13	0.50
Chromosomal Stop p.13	0.45
Chromosomal Start p.17	0.44
Chromosomal Stop p.17	0.42

The data was then split into an 80/20 training/test set, in order to train the neural network with the purpose of binary classification, therefore, to discover if the Gene concept is present in the data.

### SIENA

The final proposed framework, semi-automatic Semantic Enhancement of Datasets using Concept Recognition (SIENA), combines the two methods, gene concept recognition and property search, respectively. Using the developed method for concept recognition, the class of the dataset is identified as being gene, if the prediction is positive. In addition to this, the gene concept is used for finding the relevant properties by performing a property search in BioPortal. The list of ontologies used in the search in BioPortal can be restricted using the discovered Gene concept. The ontologies chosen are the ones that contain Gene concepts/classes (e.g HUGO, GO). AberOWL [17] is used to discover a list of appropriate ontologies, due to its ability of retrieving ontologies that use the searched term in their description. Therefore, AberOWL can be used to retrieve a list of Gene ontologies.

The list provided by AberOWL is used to perform a restricted property search in BioPortal. The discovered matches through this search are manually curated using the method previously described. The first full match is chosen for each column as property.

**Fig. 3:**
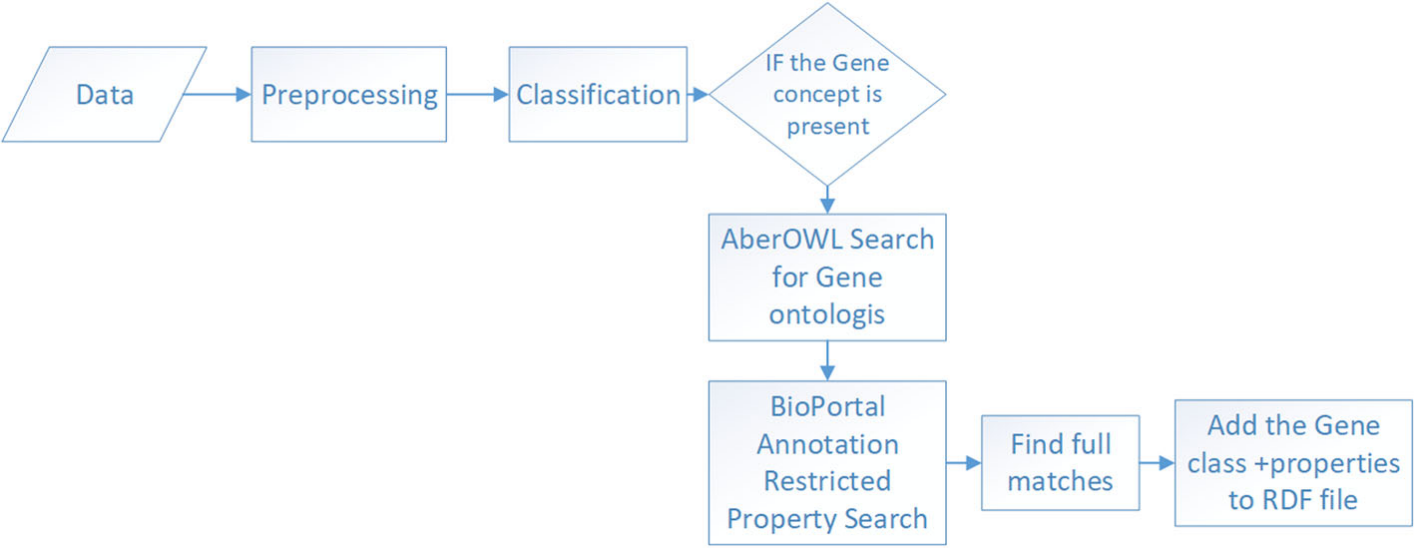
SIENA framework. Overview of the SIENA framework showing the combination of the concept recognition and restricted property search in BioPortal methods

We generate generic RDF files using the Data2Services tool. These generic RDF files are uploaded in GraphDB. The identified matches for class and properties (using SIENA) are added to the file using SPARQL update queries. The overall process is summarized in Fig. 3.

## Results

### Annotation with BioPortal

Due to a missing gold standard for the used datasets, we can not establish how semantically accurate the described methods are, rather, just in terms of its completeness in terms of finding any match. “We define a match as a term (depending on the search method used, either a class or property) found in BioPortal that could be used to replace search term from the dataset”. The results of the BioPortal search methods are evaluated using a coverage computation metric. The formula involves small variations, depending on the search method. The coverage represents the ratio between the number of columns in the dataset that was covered (replaced) by the semantic enhancement method and the total number of columns of the dataset.

#### Class search

The coverage for class search was computed using the following formula: the total number of columns which had any matches divided by the total number of columns, for each dataset. As it can be observed in Table 7, the method performs poorly, with an average coverage of 36%.

**Table 7:** Class search

Dataset	Matches	Coverage
HUGO	18	37%
CTD	4	36%
PGKB	6	35%
Average coverage		36%

#### Property search

Due to the separation of matches into the three categories (“Full Match”,“Semi-Match”,“No Match”), the coverage for this method is computed differently compared the one in the previous section. For each dataset, the coverage is computed using the formula: divide the number of columns which have any property matches as “Full Match” category from BioPortal, with the total number of columns. The results are presented in Table 8. In Table 9, the search was additionally restricted to a longest matches search (the results are the exact match of the keyword used for searching). As it can be observed from both tables, the coverage of the search without the longest match restriction is higher (40.3%) compared to the one using longest match (13%). In addition, the results for the property search are better in the case of the HGNC dataset, compared to the class search. The average coverage improved in the property search (40.3%) compared to the class search (36%).

**Table 8:** Property Search: Search performed in BioPortal with no restrictions

Dataset	Matches	Coverage
HGNC	31	63%
CTD	3	29%
PGKB	5	29%
Average coverage		40.3%

**Table 9:** LM Property Search: Restricted search using the longest match option in BioPortal

Dataset	Matches	Coverage
HGNC	13	27%
CTD	0	0%
PGKB	2	12%
Average coverage		13%

### Concept recognition

The concept recognition through binary classification model uses coverage and accuracy as metrics for the evaluation.

#### Column name

The results are presented in the confusion matrix in Table 10. The results of the classification are not high, with the accuracy reaching only 58% as its highest value. Furthermore, the classification seems biased to recognize class zero which represents the gene concept not being present. The percentages of mislabeling are fairly high (over 40%). Due to the artificial nature of the expanded dataset and the limitation of using only column names, the method performs poorly overall.

**Table 10:** Classification results using column names

True labels	Accuracy	
1	54%	46%
0	42%	58%
Predicted labels	1	0

We performed a manual analysis of false positives and false negatives over a random data sample. A common pattern in the set of false positives is column names formed of a random set of characters such as “idchromoSOMD”, “chromosoMARIANICHANAL”, “CHRGURSHACHACHALCHIC”, “GENARENACCENACCESENACE”, “acceSSioNPSEMEB”, which can resemble to column names representing. In contrast, the set of false negatives includes examples where full words are part of the column name such as “Gene_Forms”, “Gene ID”, “intermediate_filament_db”.

#### Column name and value approach

**Fig. 4:**
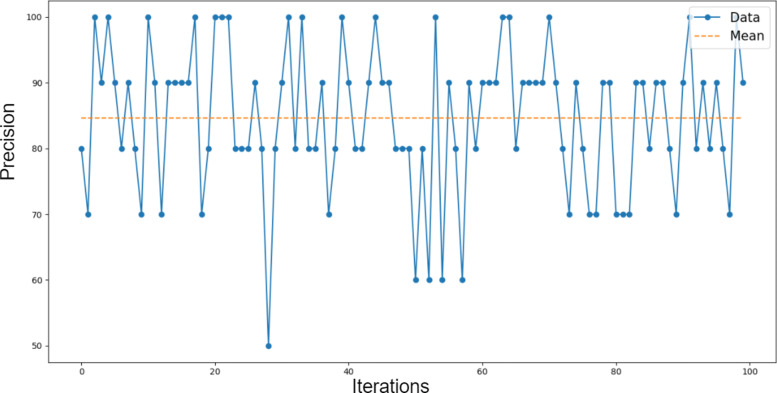
ANN precision results. Classification results using ANN; Precision over 100 test iterations

Figure 4 shows the variation of the precision during 100 test iterations, using ANN. For each separate iteration, a different random sample of the test set was selected. The method performs well, given that the precision in each test set never drops below 50%. The mean precision, considering all the performed tests is equal to 85%. This shows that both the value and column name perform well in concept recognition for a dataset.

### SIENA

The results using the proposed framework are presented in Table 11. Apart from the HGNC dataset, the coverage improved for the CTD and PGKB datasets.

**Table 11:** Framework results

Dataset	Matches	Coverage
HGNC	31	63%
CTD	8	72.7%
PGKB	7	41%
Average coverage		58.9%

In Table 12, we can observe that, compared to the other presented methods of annotation in this paper, the proposed framework achieves the highest coverage.

**Table 12:** Comparison results

Method	Average coverage
BioPortal Class Search LM	36%
BioPortal Property Search	40.3%
BioPortal Property Search LM	13%
Framework	58.9%

## Data quality assessment

Data quality is an important step in deciding the compatibility of datasets for certain tasks. Depending on the task at hand, the required data quality might be different, therefore a high data quality is not necessary for all tasks [18]. For the purpose of a thorough data quality assessment, the generic RDF files were enhanced using (i)match results from the BioPortal Property Search, and (ii) match results from the proposed framework. Therefore each dataset was converted and stored in two separate files.

### Syntactic validity

The syntactic validity of the data was measured by the errors present in the RDF syntax [19]. The used encoding format, chosen through the Data2Services tool [2], was N-Quads. During the uploading of the graphs on GraphDB^11^ (which is part of the Data2Services framework), they were checked for syntax errors. The generic generated files did not present any syntax errors. Likewise, the modification of the graphs through SPARQL queries using the collected terminology (either from the BioPortal Search or the framework), did not cause any syntax errors. Table 13 presents the datasets overview collected from GraphDB. Since the number of instances, classes and properties do not change while using the two methods the numbers are consistent, varying by dataset only.

**Table 13:** GraphDB data overview

Dataset	Class	Properties	Instances
HGNC	1	49	10858
CTD	1	11	65535
PGKB	1	17	26994

### Semantic accuracy

The semantic accuracy was analyzed through different types of issues, concerning the reasoning aspect, one of which is described in [19], specifically Ontology Hijacking. Apart from this issue two others considerable ones are added due to being present in the analyzed datasets and relevant for semantic validity: (i) broad terminology and (ii) poorly defined terminology from public ontologies. Table 14 presents the results for each resultant dataset from the two approaches (property search in BioPortal and final framework). Broad terminology refers to properties found in public ontologies with a broad description. The property “identifier”^12^ has the following definition: “Recommended best practice is to identify the resource by means of a string conforming to a formal identification system.”. There are no other constraints on how or where this property should be used. Therefore, both “gene id” and “organism id” columns, belonging to the same dataset, fit with the matched description. If the property is used in both cases in the same dataset, there will be no distinction between the types. As a result, instead of gaining semantic meaning, the data is loosing a part of it.

**Table 14:** Data quality; Columns :Broad terminology (BT), Ontology Hijacking (OH), Undefined terminology (UT), Missing definition (MD), Total Added properties (TAP), Total Properties (TP)

Dataset	BT	OH	UT	MD	TAP	TAP
CTD Property Search	3	0	8	0	3	11
CTD Framework	3	0	4	0	7	11
PGKB Property Search	3	1	12	1	5	17
PGKB Framework	0	0	0	2	7	17
HGNC Property Search	0	0	18	0	31	49
HGNC Framework	3	0	18	0	31	49

Ontology hijacking, described in [19], is defined as the usage of a property (or class) contrary to its description, thus, affecting the reasoning process. These are presented in the column Ontology Hijacking in the Table 14. As an example, property “symbol”^13^ has its range defined as: parameters, species, species reference or compartment. However, its role in the HGNC dataset was to represent gene symbols.

The error of using undefined classes and properties is presented in column Undefined terminology in Table 14. This column refers to the terms that were created by Data2Services during conversion, using column names to create properties. Whenever the tested method could not find a new suitable match to replace the generic property, the original ones were preserved. As these properties were automatically created by Data2Services, they do not exist in any public ontology.

The lack of definition in some properties, or even any other information, besides their label, makes some of the retrieved properties, hard to assess and use. Although they are part of a publicly available ontology and have a suitable name for the task, assessing their correct usage seems impossible. These are presented in column Missing definition.

### Completeness

Property completeness, described in [18], was computed as the total number of full matches divided by the total number of matches. This was computed individually for each dataset and for both annotation methods. The results are presented in Table 15. In two out of the three datasets, the trend followed in general is that completeness is increased when the SIENA framework is used. Even though the property search method retrieved more matches, the majority of them were classified as “No Matches”. Using the framework method, the relevancy of the search is increased, therefore the gap between the “Full matches” and the total number of resulting matches is narrowing, improving the semantic accuracy.

**Table 15:** Completeness results

Dataset	Completeness
CTD Property Search	22%
CTD Framework	12.8%
PGKB Property Search	2.5%
PGKB Framework	13.3%
HGNC Property Search LM	14.28%
HGNC Framework	17.94%

## Discussion

Although annotation through a biomedical ontology repository might seem sufficient to find appropriate matches when annotating a dataset, the method showed poor results on its own in our experiments. Unrestricted search on the portal generated an unfeasible number of results to be analyzed. Therefore, restrictions through several parameters were introduced. Restricted search by providing an appropriate ontology list performed best. As ontology restriction is an important step in finding the most relevant matches, the restriction approach plays a crucial role in the matches’ search. The paper introduces restriction with regard to the datatset’s concept, through concept recognition prior to the search. Neural Network Embeddings have proven to be successful for concept recognition through classification on heterogeneous data. In addition, during the training process it was discovered that the training resulted in embeddings that could be used as a separate method to provide column similarity between different columns belonging to separate datasets. However, the evaluation results show that data quality issues are present when involving public ontologies terminology. This can affect the data quality of the dataset created using the framework’s results.

The limitations imposed by the chosen methods are reflected in the selection of the input data. At the moment, the classification model needs retraining in case the data contains different attributes (not a Gene concept). Therefore, data with unseen fields and types is unsuitable for the model. In addition to the limited training, the model performs binary classification of recognition of only the gene concept, therefore other concepts can not be recognized in the same dataset. The results of the methods are missing a gold standard to be compared to, therefore their accuracy can not be established as such.

Future work will explore additional training data to allow the model to perform multi-classification (allowing multiple concepts to be recognized), in order to cover more biomedical concepts. Different methods of expansion could be used for the artificial dataset (e.g synonyms from WordNet^14^). Including the discovered measure for column similarity in the framework might benefit the annotation process, as similar columns could use the same terminology. The measure could also be included in a different framework that provides dataset comparisons.

## Conclusion

In order to answer the proposed research question, a novel semi-automated framework, SIENA, for semantic enhancement of RDF data is described in the paper. Using concept recognition combined with deep learning techniques, which reach a mean accuracy of 85%, and a biomedical ontology repository, SIENA achieves the highest results when compared to methods using only the BioPortal annotator for semantic enhancement of data. The proposed framework helps generic generated datasets to reach their full potential of providing semantically meaningful information.

## Data Availability

The SIENA framework, together with the generated and analysed data, are available for download at: http://github.com/MaastrichtU-IDS/semantic-enhancement. Declarations

## Abbreviations

ANN: Artificial Neural Network. 5, 6, 9
BT: Broad terminology. 11
CSV: Comma-separated values. 1, 2
CTD: Comparative toxicogenomics database. 3, 4, 6, 10, 11
GO: Gene ontology. 5
HGNC: Hugo gene nomenclature. 3–7, 10, 11
HUGO: Human genome organisation. 5, 10
LM: Longest matches. 3, 11
MD: Missing definition. 11
NIFSTD: Neuroscience information framework standard ontology. 4
NNE: Neural network with embeddings. 5
OH: Ontology hijacking. 11
ORTH: Orthology ontology. 4
PGKB: Pharmacogenomics knowledgebase. 3, 4, 6, 10, 11
RDF: Resource description framework. 1, 2, 7, 8
RNN: Recurrent neural network. 4, 5
SIENA: Semi-automatic semantic enhancement of datasets using concept recognition. 5–9
TAP: Total added properties. 11
TP: Total Properties. 11
TSV: Tab-separated values. 1
UT: Undefined terminology. 11
XML: extensible markup language. 1, 2

## Footnotes

1 Specification available at: https://www.w3.org/RDF/, accessed on 12 January 2019

2 Available at: https://www.genenames.org/, accessed on 10 December 2019

3 Available at: http://ctdbase.org/, accessed on 10 December 2019

4 Available at: https://www.genenames.org/, accessed on 10 December 2019

5 Available at: http://ctdbase.org/, accessed on 10 December 2019

6 Available at: https://www.pharmgkb.org, accessed on 10 December 2019

7 Available at https://bioportal.bioontology.org/, accessed on 11 January 2019

8 BioPortal Rest API available at: http://data.bioontology.org/documentation, accessed on 11 January 2019

9 Model: Laptop Dell Inspiron 5570, Intel processor i7 7th generation, 8 GB RAM

10 https://github.com/WillKoehrsen/wikipedia-data-science/blob/master/notebooks/Book%20Recommendation%20System.ipynb, accessed on 15 November 2018

11 Available for download at: https://www.ontotext.com/free-graphdb-download, accessed on 11 January 2019

12 Ontology available at: http://dublincore.org/2012/06/14/dcelements#identifier, accessed on 11 January 2019

13 Ontology available at: http://identifiers.org/biomodels.vocabulary#symbol, accessed on 11 January 2019

14 Available at: https://wordnet.princeton.edu/, accessed on 12 January 2019
